# Understanding the oxidation mechanism of methanesulfinic acid by ozone in the atmosphere

**DOI:** 10.1038/s41598-018-36405-0

**Published:** 2019-01-23

**Authors:** Guochun Lv, Chenxi Zhang, Xiaomin Sun

**Affiliations:** 1 Environment Research Institute, Shandong University, Jinan, 250100 China; 2 College of Biological and Environmental Engineering, Binzhou University, Binzhou, 256600 China

## Abstract

Methanesulfinic acid (MSIA) is an important intermediate in the oxidation of dimethyl sulfide (DMS) in the marine boundary layer. The oxidation of MSIA by ozone in the gas phase to form methanesulfonic acid (MSA) was investigated using theoretical calculations in this paper. Three pathways can be found for the reaction of MSIA with ozone. The highest energy barrier is 13.02 kcal mol^−1^ in the most favorable pathway. By comparing the reaction rate of MSIA + O_3_ with that of MSIA + OH, it can be concluded that the oxidation of MSIA by O_3_ to form MSA is of minor significance relative to its oxidation by OH radical in the gas phase. This study can provide some information for the theoretical and experimental studies in the significantly heterogeneous and aqueous-phase oxidation of MSIA by O_3_.

## Introduction

Dimethyl sulfide (CH_3_SCH_3_, DMS) emitted by oceans is the main natural source of S-containing compound^1–3^. The oxidation of dimethyl sulfide is considered of great importance in the marine boundary layer, because the main oxidation products, methanesulfonic acid (MSA) and sulfuric acid, can contribute to the formation of non-sea salt sulfate (nss-SO_4_^2−^) aerosol, which can act as the cloud condensation nuclei and can promote the formation of marine stratus clouds^4–6^. The reactions with OH radical during the day time^5,7^ and with NO_3_ radical^5,8,9^ in the night initiate the DMS oxidation, and these reactions can be classified as adduct reactions and abstraction reactions. Numerous intermediates such as dimethyl sulfoxide (CH_3_SOCH_3_: DMSO), methanesulfinic acid (CH_3_S(O)OH: MSIA), dimethyl sulfone (CH_3_SO_2_CH_3_: DMSO_2_) and CH_3_SO_x_(x = 0−3) radicals can be formed through the subsequent reaction^5,10^. The branching and many intermediates indicate that the DMS oxidation mechanism is complicated. Therefore, although a number of studies have worked on this area^5,6,10–19^, the oxidation mechanism still cannot be understood completely.

MSIA, an important intermediate in the process of DMS oxidation, is formed through the OH radical addition to DMSO with the elimination of CH_3_ radical. Using LFP-TDLAS technique, Urbanski *et al*.^20^ have proved that the reaction of DMSO with OH radical can form MSIA and CH_3_ radical in high yield. The same conclusion also is obtained in subsequent researches including laboratory studies and quantum chemistry calculations^21–27^. Barnes *et al*.^5^ also concluded that the MSIA is an important DMS secondary oxidation product through OH radical oxidation of DMSO in their review paper.

In addition to the formation of MSIA, their further oxidation also needs to be focused on. The MSIA is soluble in water, and its Henry’s law coefficient is higher than that of DMSO and lower than that of MSA^5^. Thus, the reaction involving MSIA can occur in gas phase, in aqueous phase and on the surface of aqueous atmospheric media. The OH radical and O_3_ are thought as the main oxidants in oxidizing the MSIA^5,6,18,28,29^. The ab initio and density functional theory (DFT) calculations are useful tools for mechanism research. Tian *et al*.^28^ have used DFT method to study the reaction of OH radical with methanesulfinic acid (MSIA). They found that the association-decomposition reaction (MSIA + OH → adduct → CH_3_SO_2_ + H_2_O → CH_3_ + SO_2_ + H_2_O) is more favorable than direct CH_3_ radical-abstraction reaction (MSIA + OH → adduct → CH_3_ + H_2_SO_3_ → CH_3_ + SO_2_ + H_2_O). In another theoretical study^29^, the author also found the two kinds of reaction mechanism, and concluded that the formation channel of CH_3_ radical and H_2_SO_3_ should not be negligible because more than 30% of MSIA react with OH via this channel within the range of 298–600 K.

For the oxidation of MSIA by O_3_, some studies have considered it. Enami *et al*.^18^ have performed the experiment to investigate the OH radical-initiated oxidation of DMSO on the air-water interface. In their work, they have concluded that the MSIA (or dissociated methanesulfinic acid) formed by the reaction OH + DMSO can further be oxidized by O_3_ or OH + O_2_, producing the MSA. In the modeling study of Hoffmann *et al*.^6^, the reaction of MSIA and dissociated methanesulfinic acid with O_3_ leading to MSA in the aqueous phase contribute in total 42% to MSIA degradation. However, to our knowledge, the mechanism of the reaction MSIA + O_3_ has not been considered by the theoretical study.

In this paper, we will investigate the reaction of MSIA with ozone using DFT method and ab initio method. To find the most favorable pathway, the reaction energy barrier in different pathways is calculated and compared. We also compared rate constants of the MSIA + O_3_ reaction with that of the MSIA + OH reaction, so as to evaluate their atmospheric importance.

## Results and Discussion

### The reaction of MSIA with O_3_

**Figure 1:**
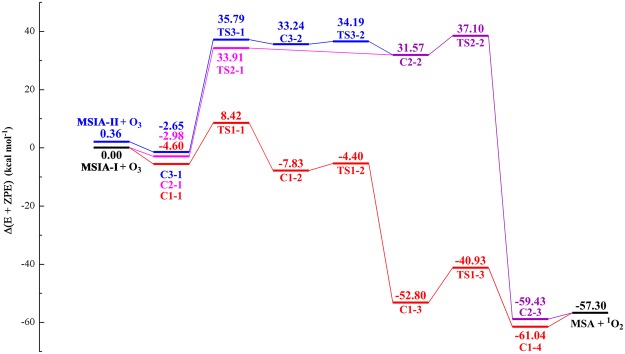
The calculated potential energy profile for the reaction of MSIA with ozone at the CCSD(T)/aug-cc-pV(T + d)Z//M06-2X/aug-cc-pV(T + d)Z level.

**Figure 2:**
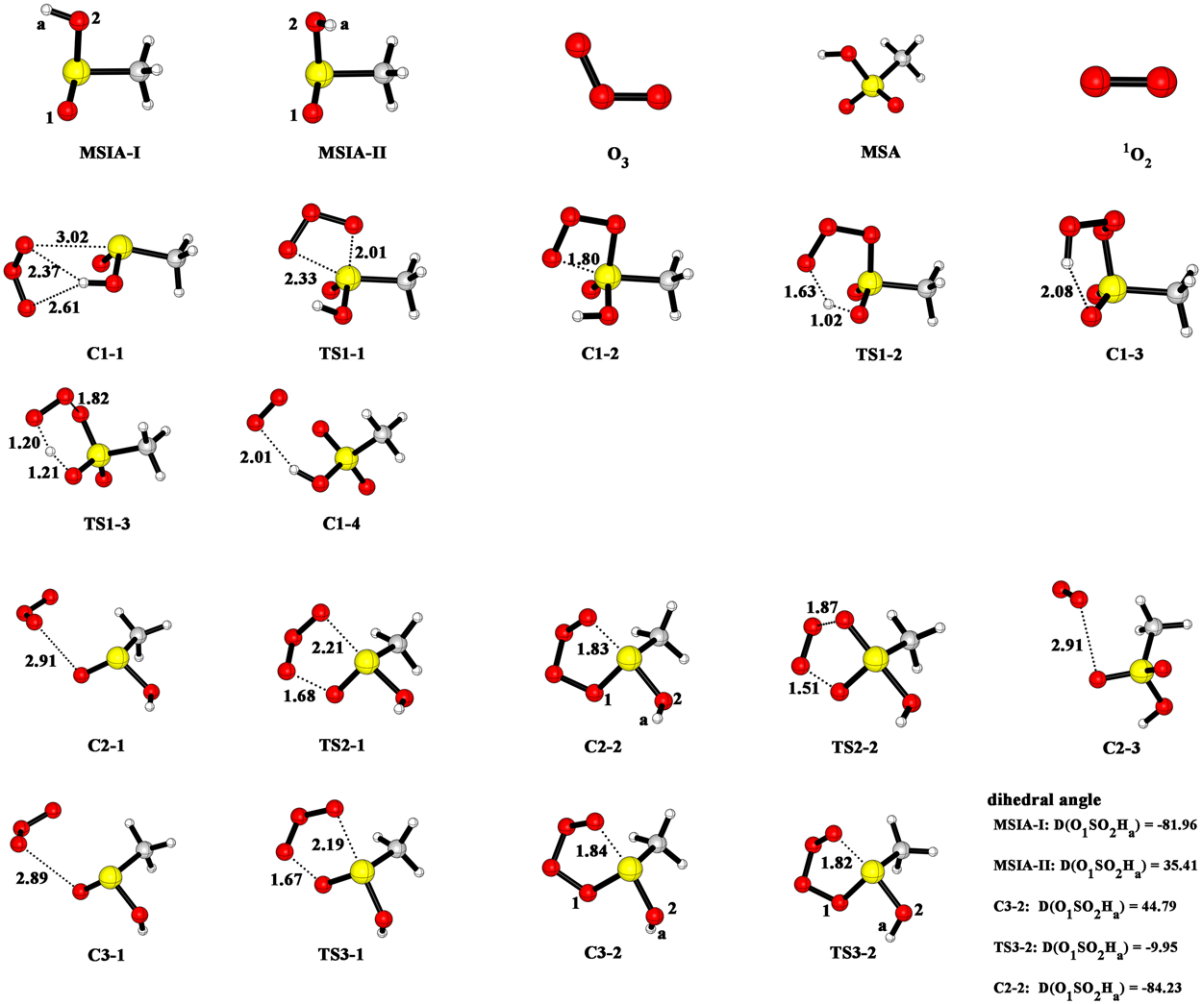
Optimized structures of reactants, complexes, transition states and products for the reaction of MSIA with ozone at the M06-2X/aug-cc-pV(T + d)Z level.

Figure 1 shows the potential energy profile for MSIA + O_3_ reaction. The corresponding structures of reactants, intermediates, transition states and products are drawn in Fig. 2. The thermodynamic data for the reaction MSIA with O_3_ are summarized in Supplementary Information (Table S1). For MSIA, as shown in Fig. 1 and Fig. 2, two conformers, called as MSIA-I and MSIA-II, can be found. The MSIA-I can transform into MSIA-II with a transition state (energy barrier of 1.00 kcal mol^−1^), which is depicted in Supplementary Fig. S1. Two pathways are identified in the reaction of MSIA-I and O_3_ (path 1: starting from C1-1, path 2: from C2-1 to MSA + ^1^O_2_). In path 1, as MSIA-I approaches the O_3_ molecule, the complex C1-1 can be formed. The C1-1 is stabilized by 4.60 kcal mol^−1^ with respect to MAIA-I + O_3_. The complex C1-1 is held together by two hydrogen bonds and one van der Waals interaction. As shown in Fig. 2, the hydrogen atom of MSIA-I interacts with the two oxygen atoms of ozone to form the two hydrogen bonds, whereas sulfur atom is involved in the formation of van der Waals interaction with an oxygen atom of ozone. After the formation of the complex C1-1, the reaction proceeds via a transition state (TS1-1) with the energy barrier of 13.02 kcal mol^−1^ related to C1-1 to form the C1-2. In the complex C1-2, one oxygen atom has bonded with sulfur atom. The subsequent transformation from C1-2 to C1-3 corresponds to the hydrogen atom transfer process. The energy barrier with the transition state TS1-2 is 3.43 kcal mol^−1^. For C1-3, it is obviously more stable than C1-2 because its energy is lower 44.97 kcal mol^−1^ than that of C1-2. The result indicates that the effect of hydrogen atom transfer process is to adjust the structure so as to increase the stability of complex. The C1-3 passes through a transition state TS1-3 with energy barrier of 11.87 kcal mol^−1^ to form the complex consisting of MSA with ^1^O_2_ (C1-4), which is lie 3.74 kcal mol^−1^ below the final product (MSA + ^1^O_2_).

In path 2, the reaction begins with the formation of complex C2-1 with the binding energy of 2.98 kcal mol^−1^. The C2-1 involves one van der Waals interaction between an oxygen atom of MSIA-I and an oxygen atom of ozone. The complex C2-2 can be formed through a transition state TS2-1 with the energy barrier of 36.89 kcal mol^−1^. Then, the C2-2 transform to the complex (C2-3) via a transition state TS2-2 with the energy barrier of 5.53 kcal mol^−1^. The TS2-2 involves the breaking of O-O bond and simultaneously the formation of O-S bond. The C2-3 is held together by one van der Waals interaction between an oxygen atom of methanesulfonic acid (MSA) and an oxygen atom of ^1^O_2_, and is expected to be stable toward break up to MSA + ^1^O_2_.

In the reaction between MSIA-II and O_3_, only one pathway (named as path 3) can be found. As the similar structure between MSIA-I and MSIA-II, its sequence of steps C3-1 → TS3-1 → C3-2, has the same feature as the sequence C2-1 → TS2-1 → C2-2. The binding energy of C3-1 relative to MSIA-II and ozone is 3.01 kcal mol^−1^. The barrier height in the process for path 3 is 38.44 kcal mol^−1^. After the complex (C3-2) formation, the reaction proceeds through transforming the dihedral angle O_1_SO_2_H_a_ to C2-2. The transformation process needs to go through a transition state TS3-2, and overcome the energy barrier of 0.95 kcal mol^−1^. Then the reaction goes on along the sequence of steps C2-2 → TS2-2 → C2-3.

To sum up, there are three pathways in the reaction of methanesulfinic acid (MSIA) with ozone. For path 2 and path 3, the initial step needs to overcome the energy barrier of around 40 kcal mol^−1^, and the energy for most of complexes and transition states is 30 kcal mol^−1^ higher than that of reactants. All barrier heights in path 1 do not exceed 14 kcal mol^−1^, and only the energy of TS1-1 is 8.42 kcal mol^−1^ higher than that of reactants. Thus, the path 1 is the most favorable reaction pathway.

### Atmospheric implications

To evaluate the importance of the methanesulfonic acid (MSA) formation by the reaction MSIA + O_3_, we will compare the rate between the reaction of MSIA with ozone and the reaction of MSIA with OH radical. Considering that the channel in which the CH_3_ radical and H_2_SO_3_ are formed accounts for about 1/3 for the reaction of MSIA with OH radical, we will compare this slower reaction channel with the favorable channel (path 1) in the reaction of MSIA with ozone.

**Figure 3:**
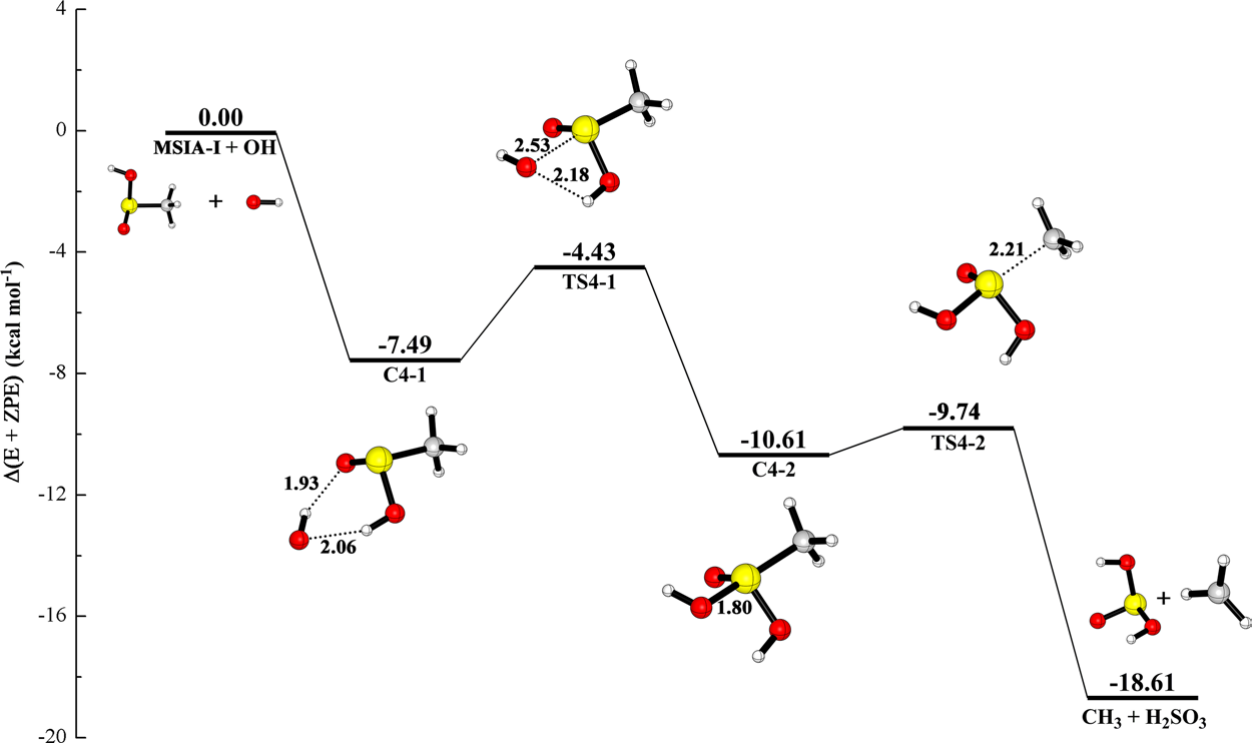
The calculated potential energy profile for the reaction of MSIA with OH radical at the CCSD(T)/aug-cc-pV(T + d)Z//M06-2X/aug-cc-pV(T + d)Z level.

Although the channel mentioned above in the reaction MSIA + OH has been reported, we also recalculate it at the CCSD(T)/aug-cc-pV(T + d)Z//M06-2X/aug-cc-pV(T + d)Z level to perform a fully legitimate comparison at the same level of theory. Figure 3 shows the potential energy and the structure of all stationary points for MSIA + OH reaction. The thermodynamic data are tabulated in Supplementary Information (Table S2). For this channel, we found that only MSIA-I can react with OH radical to form CH_3_ radical and H_2_SO_3_, which is called as path 4 in this paper. The complex (C4-1) consisting of MSIA-I and OH radical is firstly formed with the binding energy of 7.49 kcal mol^−1^ in this channel, which agrees with the value (8.14 kcal mol^−1^) calculated by González-García *et al*.^29^. Then, the C4-1 can evolve via TS4-1 with the energy barrier of 3.06 kcal mol^−1^, into the complex C4-2 whose energy is 10.61 kcal mol^−1^ lower than that of reactants. Once the C4-2 is formed, it can be easily transformed with a transition state TS4-2 into CH_3_ radical and H_2_SO_3_. The barrier height in the transformation from C4-2 to products is only 0.87 kcal mol^−1^. The barrier for the transformation from C4-1 to C4-2 is also in agreement with 3.39 kcal mol^−1^ in the literature^29^. The binding energy of C4-2 in our calculation is slightly higher than the value (12.40 kcal mol^−1^) obtained by González-García *et al*.^29^. As for the energy barrier connecting C4-2 and products, our calculated result may be more accurate than that in the mentioned literature because the value in this literature is −0.01 kcal mol^−1^. These results obtained from comparison about the reaction MSIA + OH help us to conclude that the difference between our calculation and the literature is slight.

As for path 1 (considering C1-4 as the end in kinetics analysis) and path 4, the main reaction step can be expressed as equation (1) and equation (2) in the follows:

1 $$\mathrm{MSIA} \mbox{-} {\rm{I}}+{{\rm{O}}}_{{\rm{3}}}\to \mathrm{C1} \mbox{-} 1\mathop{\longrightarrow }\limits^{TS1-1}\mathrm{C1} \mbox{-} 2\mathop{\longrightarrow }\limits^{TS1-2}\mathrm{C1} \mbox{-} 3\mathop{\longrightarrow }\limits^{TS1-3}\mathrm{C1} \mbox{-} 4$$

2 $$\mathrm{MSIA} \mbox{-} {\rm{I}}+{\rm{OH}}\to \mathrm{C4} \mbox{-} 1\mathop{\longrightarrow }\limits^{TS4-1}\mathrm{C4} \mbox{-} 2\mathop{\longrightarrow }\limits^{TS4-2}{{\rm{CH}}}_{{\rm{3}}}+{{\rm{H}}}_{{\rm{2}}}{{\rm{SO}}}_{{\rm{3}}}$$

Assuming the complex C1-1 and C4-1 is in equilibrium with their corresponding reactants, and the steady state approximation is applied, the reaction rates can be formulated as:

3 $${v}_{1}={K}_{eq1}{k}_{uni1}[\mathrm{MSIA} \mbox{-} {\rm{I}}][{{\rm{O}}}_{{\rm{3}}}]={k}_{1}[\mathrm{MSIA} \mbox{-} {\rm{I}}][{{\rm{O}}}_{{\rm{3}}}]$$

4 $${v}_{4}={K}_{eq4}{k}_{uni4}[\mathrm{MSIA} \mbox{-} {\rm{I}}][{\rm{OH}}]={k}_{4}[\mathrm{MSIA} \mbox{-} {\rm{I}}][{\rm{OH}}]$$

Where ν represents the reaction rate, *K*_*eq*_ is the equilibrium constant for the formation of C1-1 (or C4-1) from the corresponding reactants; *k*_*uni*_ is the rate constant of unimolecular rearrangement, and the subscript 1 or 4 represents path 1 or path 4.

As the multiple transition states occur in path 1 and path 4, the unified statistical model^30^ is applied to calculate k_uni_.

5 $$\frac{1}{{k}_{uni1}}=\frac{1}{{k}_{TS1-1}}+\frac{1}{{k}_{TS1-2}}+\frac{1}{{k}_{TS1-3}}$$

6 $$\frac{1}{{k}_{uni4}}=\frac{1}{{k}_{TS4-1}}+\frac{1}{{k}_{TS4-2}}$$

The relative rate for path 1 and path 4 can be obtained as follows:

7 $$\frac{{v}_{1}}{{v}_{4}}=\frac{{K}_{eq1}{k}_{uni1}[\mathrm{MSIA} \mbox{-} {\rm{I}}][{{\rm{O}}}_{{\rm{3}}}]}{{K}_{eq4}{k}_{uni4}[\mathrm{MSIA} \mbox{-} {\rm{I}}][{\rm{OH}}]}=\frac{{K}_{eq1}{k}_{uni1}[{{\rm{O}}}_{{\rm{3}}}]}{{K}_{eq4}{k}_{uni4}[{\rm{OH}}]}=\frac{{k}_{1}[{{\rm{O}}}_{{\rm{3}}}]}{{k}_{4}[{\rm{OH}}]}$$

The rate constant *k*_1_, *k*_4_ and the ratio between *k*_1_ and *k*_4_ within the range of 220 K to 298 K are shown in Table 1. The corresponding *K*_*eq*_, *k*_*TS*_ and *k*_*uni*_ are put into Supplementary Information (Table S3). As shown in Table 1, the rate constant of path 1 changes from 1.22 × 10^−24^ to 1.79 × 10^−22^ cm^3^ molecule^−1^ s^−1^ within the range of 220 to 298 K, whereas that of path 4 is transformed from 3.87 × 10^−9^ cm^3^ molecule^−1^ s^−1^ at 220 K to 2.84 × 10^−10^ cm^3^ molecule^−1^ s^−1^ at 298 K. It is obvious from the ratio between *k*_1_ and *k*_4_ that the rate constant for the reaction MSIA + O_3_ is about 10^13^–10^16^ times lower than that for the reaction MSIA + OH within the range of 220 K to 298 K. The concentration of ozone in the troposphere is a few tens of parts per billion by volume (ppbv) in mix ratio^31^. After converting the mix ratio to concentration in molecules cm^−3^ (10 ppbv = 2.50 × 10^11^ molecules cm^−3^ at 1 atm and 298 K), the concentration of ozone is ~10^11^ molecules cm^−3^. The concentration of OH radical is ~10^6^ molecules cm^−3^ in the troposphere^32^. The concentration ratio between ozone and OH radical is about 10^5^. Thus, considering the rate constants and concentration, the reaction rate of MSIA with ozone is 10^8^–10^11^ times slower than that of MSIA with OH within the range of 220–298 K. The conclusion can be drawn that the gas-phase oxidation of MSIA by ozone to form MSA is of minor importance relative to the reaction of MSIA with OH radical. The result also indicates that the oxidation of DMSO through MSIA intermediate in the gas phase mainly produce SO_2_ (subsequently converting to H_2_SO_4_).

**Table 1:** The rate constant (cm^3^ molecule^−1^ s^−1^) and rate constant ratio for the reaction of MSIA with ozone and with OH radical.

T (K)	220	240	260	280	298
k1	1.22 × 10^−24^	5.88 × 10^−24^	2.24 × 10^−23^	7.13 × 10^−23^	1.79 × 10^−22^
k2	3.87 × 10^−9^	1.67 × 10^−9^	8.21 × 10^−10^	4.51 × 10^−10^	2.84 × 10^−10^
k1/k2	3.15 × 10^−16^	3.53 × 10^−15^	2.73 × 10^−14^	1.58 × 10^−13^	6.31 × 10^−13^

## Conclusion

The oxidation of methanesulfinic acid (CH_3_S(O)OH: MSIA) by ozone in the atmosphere has been investigated in this paper using the quantum chemical calculations. Two conformers of MSIA and the three reaction pathways can be found. The pathway (MSIA-I + O_3_ → C1-1 → TS1-1 → C1-2 → TS1-2 → C1-3 → TS1-3 → C1-4 → MSA + ^1^O_2_) is the most favorable because its highest energy barrier is 13.02 kcal mol^−1^, which is evidently lower than those of another two pathways. By kinetics analysis, the oxidation of MSIA by ozone is 8–11 orders of magnitude slower than that by OH radical in the range of 220–298 K. The result indicates that the oxidation of MSIA by ozone is minor important in most cases relative to its oxidation by OH radical in the gas phase. Although it has shown the unimportance of the reaction in the gas phase, the study about the gas-phase oxidation of MSIA by ozone is meaningful because it can provide some information for the theoretical and experimental studies in the significantly heterogeneous and aqueous-phase oxidation of MSIA by ozone.

## Methods

All geometric structures were optimized using M06-2X method^33^ with the aug-cc-pVTZ basis set^34^ for the H, C, O atoms and aug-cc-pV(T + d)Z basis set^35^ for the S atom (for simplification, the group of basis set was called as aug-cc-pV(T + d)Z). For aug-cc-pV(T + d)Z basis set, it is to add one additional high-exponent d function to aug-cc-pVTZ so as to obtain satisfactory convergence behavior^35^. The revised basis set has been used in many sulfur-containing systems, and it has been proved that the aug-cc-pV(T + d)Z basis set can obtain more accurate results^36–38^. After optimization, the frequency calculation was performed in order to confirm the energy minimum points and transition states, and to obtain the zero point energy correction and thermal correction. The intrinsic reaction coordinate (IRC) calculation^39–41^ also was carried out to ensure that the transition states connected with the corresponding reactants and products. For M06-2X method, the ultrafine integration grid was chosen to enhance calculation accuracy at reasonable additional cost. Single-point energies were refined using the CCSD(T) method^42,43^ with aug-cc-pV(T + d)Z basis set. To estimate the extent of nondynamic correlation, the T_1_ diagnostic (computed using CCSD(T)/aug-cc-pV(T + d)Z) was used^44^. If the value of T_1_ is more than 0.04, the single reference wave function is considered to be unreliable^45^. The value of T_1_ calculated for all species in this paper (see Supplementary Table S4) is below 0.04, indicating that the CCSD(T) method is suitable. All quantum chemistry calculations were carried out with Gaussian 09 suit of software^46^. The geometries were drawn using the CYLview software package^47^.

For the reaction of MSIA with O_3_, it involves in the O_3_ of biradical character and produced ^1^O_2_, which makes the optimization process more complex. It is because for biradical stationary points the unrestricted formalism needs to be considered so as to obtain the stable broken-symmetry solution. Our strategy is firstly to use the restricted M06-2X method to gain all stationary points in these pathways, then to verify the stability of the wave function using the keyword *stable* in Gaussian. It can be found that there are two kinds of stationary points related to the unstable wave function: one is from the beginning of pathway, which consists of O_3_-MSIA complex and the first transition state in the pathway; another is from the end of pathway, which composes of the last transition state and produced ^1^O_2_-MSA complex. For the former, the reason of wave function instability is biradical character of O_3_. We re-optimize these points by specifying the keyword *stable* = *opt* to generate a stable initial guess. After the geometries were optimized, the stability of wave function is tested again using the keyword *stable*. If the instability is found, it needs to repeat the optimized process until the wave function is stable. For the letter, the wave function instability is understandable because the ground state of O_2_ is a triple state. Since the MSIA with O_3_ react in the singlet potential energy surface, the produced O_2_ is singlet state. Thus, in this case, the stationary points related to wave function instability do not need to re-optimize.

For the kinetics analysis, the electronic energies based on the CCSD(T)/aug-cc-pV(T + d)Z level of theory, while the partition functions obtained from the M06-2X/aug-cc-pV(T + d)Z level of theory. The conventional transition-state theory (TST)^48^ with Wigner tunneling correction was used to calculate the rate constants. All rate constants were calculated by using the KiSThelP program^49^.

## Electronic supplementary material

Supplementary Information

## Data Availability

The data generated or analyzed during the current study are available from the corresponding author on reasonable request.

## References

[1] Andreae MO (1990). Ocean-atmosphere interactions in the global biogeochemical sulfur cycle. Mar. Chem..

[2] Bates TS, Lamb BK, Guenther A, Dignon J, Stoiber RE (1992). Sulfur emissions to the atmosphere from natural sourees. J. Atmos. Chem..

[3] Andreae MO, Crutzen PJ (1997). Atmospheric Aerosols: Biogeochemical Sources and Role in Atmospheric Chemistry. Science.

[4] Charlson RJ, Lovelock JE, Andreae MO, Warren SG (1987). Oceanic phytoplankton, atmospheric sulphur, cloud albedo and climate. Nature.

[5] Barnes I, Hjorth J, Mihalopoulos N (2006). Dimethyl Sulfide and Dimethyl Sulfoxide and Their Oxidation in the Atmosphere. Chem. Rev..

[6] Hoffmann EH (2016). An advanced modeling study on the impacts and atmospheric implications of multiphase dimethyl sulfide chemistry. Proc. Natl. Acad. Sci. USA.

[7] Cooper DJ (1996). Estimation of hydroxyl radical concentrations in the marine atmospheric boundary layer using a reactive atmospheric tracer. J. Atmos. Chem..

[8] Butkovskaya NI, LeBras G (1994). Mechanism of the NO3+ DMS Reaction by Discharge Flow Mass Spectrometry. J. Phys. Chem..

[9] Nakano Y, Ishiwata T, Aloisio S, Kawasaki M (2006). Temperature and Pressure Dependence of the Rate Constants of the Reaction of NO3 Radical with CH3SCH3. J. Phys. Chem. A.

[10] Mardyukov A, Schreiner PR (2018). Atmospherically Relevant Radicals Derived from the Oxidation of Dimethyl Sulfide. Acc. Chem. Res..

[11] Yin F, Grosjean D, Seinfeld JH (1990). Photooxidation of dimethyl sulfide and dimethyl disulfide. I: Mechanism development. J. Atmos. Chem..

[12] Yin F, Grosjean D, Flagan RC, Seinfeld JH (1990). Photooxidation of dimethyl sulfide and dimethyl disulfide. II: Mechanism evaluation. J. Atmos. Chem..

[13] Hynes AJ (1995). A Mechanistic Study of the Reaction of OH with Dimethyl-d6 Sulfide. Direct Observation of Adduct Formation and the Kinetics of the Adduct Reaction with O2. J. Phys. Chem..

[14] Barone SB, Turnipseed AA, Ravishankara AR (1996). Reaction of OH with Dimethyl Sulfide (DMS). 1. Equilibrium Constant for OH+ DMS Reaction and the Kinetics of the OH·DMS+ O2 Reaction. J. Phys. Chem..

[15] Capaldo KP, Pandis SN (1997). Dimethylsulfide chemistry in the remote marine atmosphere: Evaluation and sensitivity analysis of available mechanisms. J. Geophys. Res-Atmos..

[16] Gross A, Barnes I, Sørensen RM, Kongsted J, Mikkelsen KV (2004). A Theoretical Study of the Reaction between CH3S(OH)CH3 and O2. J. Phys. Chem. A.

[17] Jørgensen S, Kjaergaard HG (2010). Effect of Hydration on the Hydrogen Abstraction Reaction by HO in DMS and its Oxidation Products. J. Phys. Chem. A.

[18] Enami S (2016). “Sizing” Heterogeneous Chemistry in the Conversion of Gaseous Dimethyl Sulfide to Atmospheric Particles. Environ. Sci. Technol..

[19] Domin D, Braïda B, Bergès J (2017). Influence of Water on the Oxidation of Dimethyl Sulfide by the ·OH Radical. J. Phys. Chem. B.

[20] Urbanski SP, Stickel RE, Wine PH (1998). Mechanistic and Kinetic Study of the Gas-Phase Reaction of Hydroxyl Radical with Dimethyl Sulfoxide. J. Phys. Chem. A.

[21] Arsene C, Barnes I, Becker H (1999). K. FT-IR product study of the photo-oxidation of dimethyl sulfide: Temperature and O2 partial pressure dependence. PCCP.

[22] Arsene C (2002). Formation of Methane Sulfinic Acid in the Gas-Phase OH-Radical Initiated Oxidation of Dimethyl Sulfoxide. Environ. Sci. Technol..

[23] Kukui A, Borissenko D, Laverdet G, Le Bras G (2003). Gas-Phase Reactions of OH Radicals with Dimethyl Sulfoxide and Methane Sulfinic Acid Using Turbulent Flow Reactor and Chemical Ionization Mass Spectrometry. J. Phys. Chem. A.

[24] Wang L, Zhang J (2002). Ab initio study of reaction of dimethyl sulfoxide (DMSO) with OH radical. Chem. Phys. Lett..

[25] Resende SM, de Bona JC, Sombrio PdS (2005). Theoretical study of the role of adducts in the atmospheric oxidation of dimethyl sulfoxide by OH, O2 and O3 and the kinetics of the reaction DMSO+ OH. Chem. Phys..

[26] Baptista L, da Silva EC, Arbilla G (2008). Theoretical investigation of the gas phase oxidation mechanism of dimethyl sulfoxide by OH radical. J. Mol. Struct. THEOCHEM.

[27] González-García N, González-Lafont À, Lluch JM (2006). Variational Transition-State Theory Study of the Dimethyl Sulfoxide (DMSO) and OH Reaction. J. Phys. Chem. A.

[28] Tian Y (2007). Ab initio study of the reaction of OH radical with methyl sulfinic acid (MSIA). Chem. Phys..

[29] González-García N, González-Lafont À, Lluch JM (2007). Methanesulfinic Acid Reaction with OH:  Mechanism, Rate Constants, and Atmospheric Implications. J. Phys. Chem. A.

[30] Miller WH (1976). Unified statistical model for “complex” and “direct” reaction mechanisms. J. Chem. Phys..

[31] Cooper OR (2010). Increasing springtime ozone mixing ratios in the free troposphere over western North America. Nature.

[32] Stone D, Whalley LK, Heard DE (2012). Tropospheric OH and HO2 radicals: field measurements and model comparisons. Chem. Soc. Rev..

[33] Zhao Y, Truhlar DG (2008). The M06 suite of density functionals for main group thermochemistry, thermochemical kinetics, noncovalent interactions, excited states, and transition elements: two new functionals and systematic testing of four M06-class functionals and 12 other functionals. Theor. Chem. Acc..

[34] Dunning TH (1989). Gaussian basis sets for use in correlated molecular calculations. I. The atoms boron through neon and hydrogen. J. Chem. Phys..

[35] Dunning TH, Peterson KA, Wilson AK (2001). Gaussian basis sets for use in correlated molecular calculations. X. The atoms aluminum through argon revisited. J. Chem. Phys..

[36] Wang NX, Wilson AK (2003). Effects of Basis Set Choice upon the Atomization Energy of the Second-Row Compounds SO2, CCl, and ClO2 for B3LYP and B3PW91. J. Phys. Chem. A.

[37] Wilson AK, Dunning TH (2004). The HSO−SOH Isomers Revisited:  The Effect of Tight d Functions. J. Phys. Chem. A.

[38] Long B (2012). Formic Acid Catalyzed Gas-Phase Reaction of H2O with SO3 and the Reverse Reaction: A Theoretical Study. Chem Phys Chem.

[39] Fukui K (1981). The path of chemical reactions - the IRC approach. Acc. Chem. Res..

[40] Hratchian HP, Schlegel HB (2004). Accurate reaction paths using a Hessian based predictor–corrector integrator. J. Chem. Phys..

[41] Hratchian HP, Schlegel HB (2005). Using Hessian Updating To Increase the Efficiency of a Hessian Based Predictor-Corrector Reaction Path Following Method. J. Chem. Theory Comput..

[42] Purvis GD, Bartlett RJ (1982). A full coupled-cluster singles and doubles model: The inclusion of disconnected triples. J. Chem. Phys..

[43] Pople JA, Head‐Gordon M, Raghavachari K (1987). Quadratic configuration interaction. A general technique for determining electron correlation energies. J. Chem. Phys..

[44] Lee TJ, Taylor PR (1989). A diagnostic for determining the quality of single-reference electron correlation methods. Int. J. Quantum Chem..

[45] Akbar Ali M, M B, Lin KC (2018). Catalytic effect of a single water molecule on the OH+ CH2NH reaction. PCCP.

[46] Gaussian 09, Revision B.01, Frisch, M. J. *et al*., Gaussian, Inc., Wallingford CT, 2010.

[47] Legault, C. Y. CYLview, 1.0b. *Université de Sherbrooke*, http://www.cylview.org (2009).

[48] Truhlar DG, Garrett BC, Klippenstein SJ (1996). Current Status of Transition-State Theory. J. Phys. Chem..

[49] Canneaux S, Bohr F, Henon E (2014). KiSThelP: A program to predict thermodynamic properties and rate constants from quantum chemistry results†. J. Comput. Chem..

