# Supplementary material for: Understanding the oxidation mechanism of methanesulfinic acid by ozone in the atmosphere
Source: Sci Rep. 2019 Jan 23;9:322. doi: 10.1038/s41598-018-36405-0 (PMC6344471; doi:10.1038/s41598-018-36405-0)
Supplement: Supplementary file 1 — Supplementary Information [file 41598_2018_36405_MOESM1_ESM.doc]

Supplementary Information for

**Understanding the oxidation mechanism of methanesulfinic acid by ozone in the atmosphere**

Guochun Lv^1^, Chenxi Zhang^2^, Xiaomin Sun^1,*^

^1^Environment Research Institute, Shandong University, Jinan 250100, China

^2^College of Biological and Environmental Engineering, Binzhou University, Binzhou 256600, China

^*^Corresponding authors: Xiaomin Sun, [sxmwch@sdu.edu.cn](mailto:sxmwch@sdu.edu.cn).


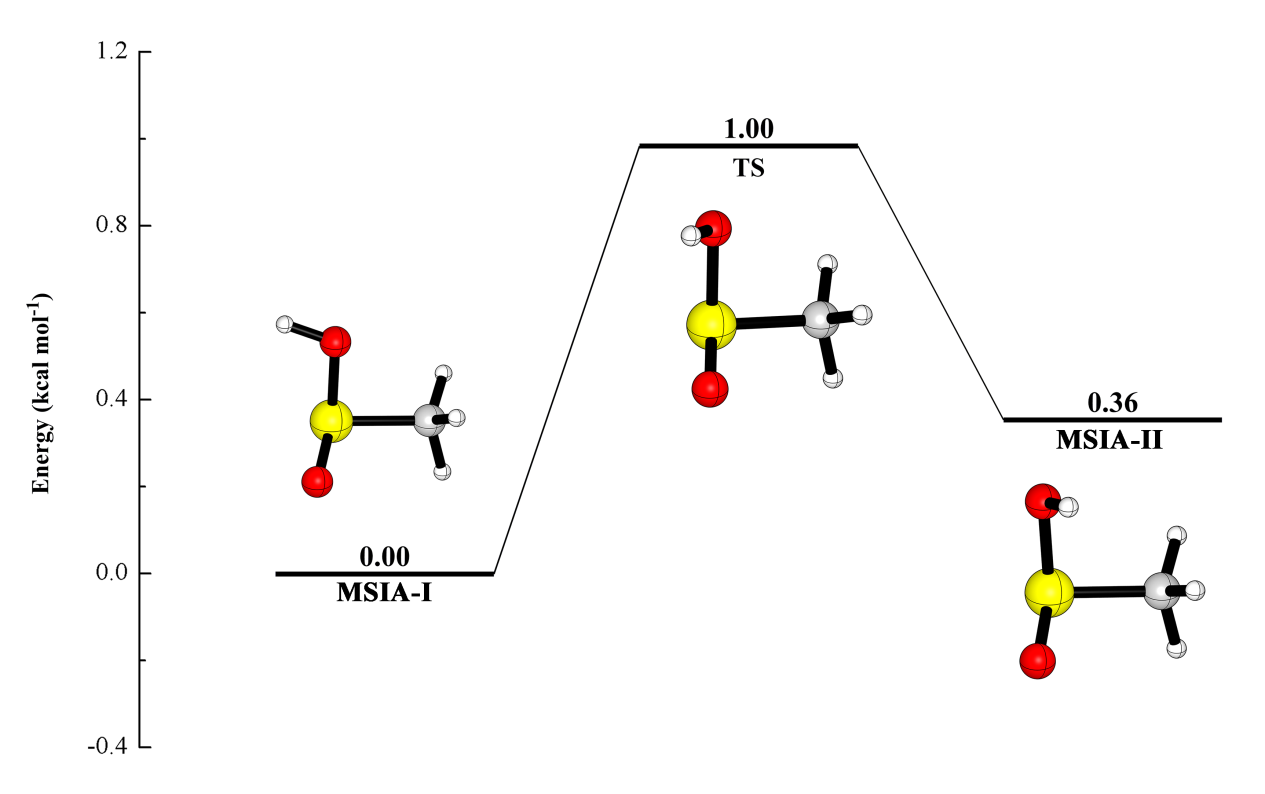


Figure S1. The calculated potential energy profile for the transformation reaction between two conformers of MSIA at the CCSD(T)/aug-cc-pV(T+d)Z//M06-2X/ aug-cc-pV(T+d)Z level.

Table S1. Energies, enthalpies and free energies for the reaction of MSIA + O_3_ (in kcal mol^-1^) at the CCSD(T)/aug-cc-pV(T+d)Z//M06-2X/ aug-cc-pV(T+d)Z level.

| species | ΔE | Δ(E+ZPE) | ΔH^a^ | ΔG^a^ |
| --- | --- | --- | --- | --- |
| MSIA-I + O_3_ | 0.00 | 0.00 | 0.00 | 0.00 |
| MSIA-II+ O_3_ | 0.58 | 0.36 | 0.35 | 0.37 |
| C1-1 | -5.60 | -4.60 | -4.26 | 5.06 |
| TS1-1 | 6.44 | 8.42 | 7.50 | 20.75 |
| C1-2 | -12.02 | -7.83 | -9.02 | 4.89 |
| TS1-2 | -6.74 | -4.40 | -5.72 | 8.36 |
| C1-3 | -57.23 | -52.80 | -53.85 | -40.32 |
| TS1-3 | -42.22 | -40.93 | -42.28 | -28.14 |
| C1-4 | -63.78 | -61.04 | -60.97 | -50.99 |
| C2-1 | -3.77 | -2.98 | -2.49 | 5.90 |
| TS2-1 | 32.23 | 33.91 | 33.06 | 45.82 |
| C2-2 | 28.62 | 31.57 | 30.53 | 44.03 |
| TS2-2 | 35.63 | 37.10 | 36.12 | 49.60 |
| C2-3 | -61.74 | -59.43 | -59.02 | -50.53 |
| C3-1 | -3.26 | -2.65 | -2.19 | 6.48 |
| TS3-1 | 34.20 | 35.79 | 34.86 | 47.93 |
| C3-2 | 30.54 | 33.24 | 32.24 | 45.62 |
| TS3-2 | 31.96 | 34.19 | 32.94 | 46.91 |
| MSA + ^1^O_2_ | -59.16 | -57.30 | -57.36 | -54.87 |

^a^The enthalpy and free energy are obtained at 298.15 K and 1 atm.

Table S2. Energies, enthalpies and free energies for the reaction of MSIA + OH (in kcal mol^-1^) at the CCSD(T)/aug-cc-pV(T+d)Z//M06-2X/ aug-cc-pV(T+d)Z level.

| species | ΔE | Δ(E+ZPE) | ΔH^a^ | ΔG^a^ |
| --- | --- | --- | --- | --- |
| MSIA-I + OH | 0.00 | 0.00 | 0.00 | 0.00 |
| C4-1 | -9.25 | -7.49 | -8.08 | 0.29 |
| TS4-1 | -6.30 | -4.43 | -5.41 | 4.03 |
| C4-2 | -14.02 | -10.61 | -11.72 | -1.57 |
| TS4-2 | -11.36 | -9.74 | -10.72 | -1.05 |
| CH_3_ + H_2_SO_3_ | -16.90 | -18.61 | -18.33 | -20.35 |

^a^The enthalpy and free energy are obtained at 298.15 K and 1 atm.

Table S3. The equilibrium constants (K_eq_, cm^3^ molecule^-1^), unimolecular rate constants (k_TS_, s^-1^) and Wigner correction coefficient (***κ_TS_***), and total unimolecular rate constants (k_uni_, s^-1^) for the reaction of MSIA with ozone (path 1) and with OH (path 4) at the different temperatures.

| T (K) | 220 | 240 | 260 | 280 | 298 |
| --- | --- | --- | --- | --- | --- |
| K_eq1_ | 8.17×10^-23^ | 3.81×10^-23^ | 2.01×10^-23^ | 1.19×10^-23^ | 7.94×10^-24^ |
| k_TS1-1_ | 1.49×10^-2^ | 1.54×10^-1^ | 1.11 | 6.02 | 2.26×10^1^ |
| ***κ_TS1-1_*** | 1.30 | 1.25 | 1.21 | 1.18 | 1.16 |
| k_TS1-2_ | 2.32×10^9^ | 4.64×10^9^ | 8.38×10^9^ | 1.39×10^10^ | 2.09×10^10^ |
| ***κ_TS1-2_*** | 1.32 | 1.27 | 1.23 | 1.20 | 1.17 |
| k_TS1-3_ | 1.62×10^1^ | 1.46×10^2^ | 9.33×10^2^ | 4.59×10^3^ | 1.60×10^4^ |
| ***κ_TS1-3_*** | 3.19 | 2.84 | 2.57 | 2.35 | 2.19 |
| k_uni1_ | 1.49×10^-2^ | 1.54×10^-1^ | 1.11 | 6.01 | 2.26×10^1^ |
| K_eq4_ | 2.38×10^-18^ | 5.54×10^-19^ | 1.62×10^-19^ | 5.68×10^-20^ | 2.52×10^-20^ |
| k_TS4-1_ | 1.63×10^9^ | 3.01×10^9^ | 5.08×10^9^ | 7.95×10^9^ | 1.13×10^10^ |
| ***κ_TS4-1_*** | 1.02 | 1.01 | 1.01 | 1.01 | 1.01 |
| k_TS4-2_ | 1.25×10^12^ | 1.61×10^12^ | 2.01×10^12^ | 2.44×10^12^ | 2.87×10^12^ |
| ***κ_TS4-2_*** | 1.19 | 1.16 | 1.14 | 1.12 | 1.10 |
| k_uni4_ | 1.63×10^9^ | 3.01×10^9^ | 5.07×10^9^ | 7.93×10^9^ | 1.13×10^10^ |

Table S4. The T1 diagnostic value for all species

| Species | T1 diagnostic value |
| --- | --- |
| MSIA-I | 0.0167 |
| MSIA-II | 0.0164 |
| O_3_ | 0.0266 |
| OH | 0.0100 |
| C1-1 | 0.0217 |
| TS1-2 | 0.0368 |
| C1-2 | 0.0163 |
| TS1-2 | 0.0191 |
| C1-3 | 0.0174 |
| TS1-3 | 0.0246 |
| C1-4 | 0.0160 |
| C2-1 | 0.0214 |
| TS2-1 | 0.0365 |
| C2-2 | 0.0200 |
| TS2-2 | 0.0375 |
| C2-3 | 0.0155 |
| C3-1 | 0.0213 |
| TS3-1 | 0.0362 |
| C3-2 | 0.0197 |
| TS3-2 | 0.0375 |
| MSA | 0.0154 |
| ^1^O_2_ | 0.0147 |
| C4-1 | 0.0157 |
| TS4-1 | 0.0218 |
| C4-2 | 0.0192 |
| TS4-2 | 0.0198 |
| H_2_SO_3_ | 0.0172 |
| CH_3_ | 0.0088 |
| TS (see Figure S1) | 0.0164 |

Table S5. Optimized geometries of all species in this paper at the the M06-2X/ aug-cc-pV(T+d)Z level.

**MSIA-I**

-0.151960 -0.154648 -0.417874

-0.664988 -1.271364 0.369793

1.522135 0.118249 0.148656

1.925699 1.009661 -0.324084

1.475711 0.226679 1.230316

2.094026 -0.764048 -0.126857

-0.664055 1.254429 0.261659

-1.564547 1.428069 -0.036948

**MSIA-II**

-0.132872 -0.147328 -0.437959

-0.616055 -1.268593 0.370280

1.524435 0.172438 0.177328

1.892483 1.103915 -0.245643

1.469450 0.219858 1.264293

2.148412 -0.664456 -0.128428

-0.834483 1.209388 0.125376

-0.926695 1.136935 1.087906

**O_3_**

0.000000 0.444862 0.000000

1.068200 -0.222432 0.000000

-1.068200 -0.222430 0.000000

**OH**

0.000000 0.000000 0.107992

0.000000 0.000000 -0.863937

**C1-1**

1.055834 -0.070554 -0.429019

0.368794 -1.208696 0.186432

2.735997 -0.123506 0.178011

3.264960 0.768806 -0.146292

2.679236 -0.181387 1.262887

3.191842 -1.019532 -0.235292

0.719581 1.273670 0.442543

-2.387449 -0.334007 -0.118434

-1.871528 0.410392 -0.996287

-2.110783 -0.044094 1.069397

-0.194284 1.523895 0.245731

**TS1-1**

0.519465 -0.161378 -0.138671

1.046716 -1.112638 -1.085900

1.739082 1.113748 0.044828

1.370455 1.843649 0.758323

2.648507 0.630835 0.393682

1.883551 1.551761 -0.938352

0.537215 -0.671229 1.356110

-1.957598 0.440857 -0.372814

-1.716735 -0.759993 0.106525

-0.942942 1.205441 0.019982

-0.381689 -0.946182 1.544897

**C1-2**

0.322876 -0.146306 -0.109627

0.706067 -0.963728 -1.220261

1.756298 0.907801 0.073178

1.647002 1.554786 0.936932

2.606860 0.242313 0.182201

1.849269 1.482736 -0.843493

0.486506 -0.745111 1.354211

-1.884968 0.589130 0.024443

-1.382441 -0.722587 -0.114901

-0.612897 1.202194 -0.108727

-0.305083 -1.264931 1.561217

**TS1-2**

0.429664 -0.147161 -0.092360

0.656955 -1.045447 -1.167879

1.937744 0.728862 0.170929

2.690361 -0.031530 0.363706

2.167048 1.261460 -0.747400

1.826108 1.404514 1.011728

0.224750 -0.704797 1.340512

-1.818796 0.751757 -0.033833

-1.669820 -0.634510 -0.051621

-0.445844 1.168922 -0.267903

-0.762575 -0.940433 1.269952

**C1-3**

0.708815 -0.210811 0.038684

1.655414 -0.930753 -0.733905

1.303148 1.404633 0.401196

2.099516 1.282907 1.131356

1.679822 1.838398 -0.520081

0.483452 1.983678 0.817037

0.120924 -0.767238 1.217910

-1.562727 0.710521 -0.287496

-2.405886 -0.298803 0.142402

-0.493886 0.097231 -1.010646

-1.933422 -0.647464 0.919453

**TS1-3**

0.683590 -0.160016 -0.046221

1.824433 -0.916936 -0.419403

1.178258 1.437871 0.496279

1.815701 1.302091 1.365664

1.728442 1.896918 -0.320527

0.289097 2.010540 0.743765

-0.096346 -0.747023 1.091816

-1.774350 0.636425 -0.345086

-2.252464 -0.360860 0.248065

-0.269631 0.058671 -1.193057

-1.293361 -0.638733 0.914277

**C1-4**

-0.887931 -0.121304 0.113718

-2.197708 -0.672086 0.088561

-0.988716 1.594027 -0.278534

-1.526224 1.696666 -1.216508

-1.537156 2.065974 0.532702

0.018982 1.993114 -0.348315

-0.114034 -0.694491 -1.146700

2.535905 0.634164 0.097994

2.609635 -0.538197 -0.069475

-0.036881 -0.292664 1.252251

0.808246 -0.872868 -0.897207

**C2-1**

-0.985291 -0.208945 -0.248264

-0.250897 -0.561792 0.967259

-1.413536 1.517255 -0.074906

-2.062357 1.812828 -0.895033

-1.911735 1.623975 0.886503

-0.480097 2.073388 -0.093633

-2.552209 -0.672645 -0.061021

2.531474 -0.036726 0.231045

1.972830 1.062398 -0.025716

2.214071 -0.998867 -0.511201

-2.622095 -1.609533 -0.279112

**TS2-1**

-0.688120 -0.024699 -0.397459

0.081371 -0.980860 0.591012

-1.037204 1.403203 0.623082

-1.432856 2.176835 -0.031131

-1.776270 1.097070 1.356656

-0.103514 1.724215 1.066784

-2.203051 -0.641814 -0.107896

2.017401 0.232567 0.219084

1.283512 0.927043 -0.690006

1.685606 -0.996321 0.109364

-2.372933 -1.347076 -0.743932

**C2-2**

-0.633722 0.073704 -0.483576

0.212003 -1.093713 0.402466

-0.950253 1.367843 0.714169

-1.106219 2.281764 0.147228

-1.822530 1.101809 1.297725

-0.056101 1.460219 1.322969

-2.024106 -0.735644 0.038576

1.822976 0.397564 0.268330

1.014185 0.817216 -0.783218

1.601754 -0.979754 0.229031

-2.188565 -1.475478 -0.557184

**TS2-2**

0.572781 0.043353 0.395292

-0.259719 -1.103663 -0.374857

0.925413 1.335949 -0.777867

1.743452 1.918502 -0.362518

1.236789 0.865420 -1.704696

0.024367 1.926234 -0.889151

2.043699 -0.713849 -0.059782

-1.904920 0.486161 -0.238510

-0.628311 0.636900 1.114894

-1.734921 -0.792199 -0.335621

2.151792 -1.526310 0.449907

**C2-3**

0.782545 -0.133583 0.024482

0.061365 -0.576345 -1.130252

0.245619 1.482215 0.474412

0.911292 1.856008 1.246778

0.261079 2.113884 -0.408563

-0.766703 1.372746 0.854571

2.276590 0.156310 -0.448913

-2.444095 -0.486967 0.355699

0.857579 -0.935208 1.195096

-2.842451 0.326753 -0.412380

2.327986 0.025063 -1.404979

**C3-1**

-0.977558 -0.199323 -0.278016

-0.271084 -0.563878 0.956103

-1.407825 1.529009 -0.054137

-1.855480 1.632556 0.933632

-0.483124 2.098618 -0.109340

-2.096491 1.831108 -0.839313

-2.481411 -0.811931 -0.174304

-2.781714 -0.752692 0.745656

2.520942 -0.028403 0.235688

1.945805 1.060978 -0.028699

2.198834 -1.006074 -0.483484

**TS3-1**

-0.689620 -0.018894 -0.414181

0.069886 -0.971648 0.589647

-1.014203 1.443021 0.587488

-2.021125 1.372529 0.987092

-0.257042 1.480156 1.363648

-0.913601 2.307408 -0.062138

-2.159467 -0.736435 -0.248353

-2.357344 -0.957970 0.673151

2.022063 0.191551 0.221913

1.256479 0.966537 -0.595847

1.644570 -1.019748 0.050167

**C3-2**

-0.644147 0.090700 -0.499227

0.195533 -1.112922 0.349070

-0.916091 1.367273 0.738441

-1.896075 1.237094 1.181840

-0.117322 1.287017 1.471340

-0.841557 2.319907 0.222815

-2.015222 -0.786160 -0.112700

-2.051186 -1.087310 0.805371

1.803983 0.388328 0.291435

1.017139 0.825595 -0.766806

1.587197 -0.991284 0.223453

**TS3-2**

-0.624169 0.110263 -0.511031

0.209277 -1.110212 0.327584

-0.980872 1.333220 0.753272

-2.019189 1.617549 0.615027

-0.862892 0.834063 1.712392

-0.304655 2.171620 0.646309

-2.015355 -0.757462 -0.064223

-1.819726 -1.620712 0.317622

1.789850 0.413464 0.314882

1.025048 0.828106 -0.770980

1.600980 -0.969652 0.238426

**MSA**

-0.086819 0.135066 0.064403

-0.219351 1.392013 -0.585156

1.600443 -0.365756 -0.018865

1.909553 -0.346146 -1.059701

2.165084 0.358514 0.562806

1.692250 -1.359889 0.407779

-0.780092 -0.955773 -0.868219

-0.582733 -0.071978 1.389249

-1.323026 -1.533101 -0.315127

**^1^O_2_**

0.000000 0.000000 0.593979

0.000000 0.000000 -0.593979

**C4-1**

0.479913 -0.127657 -0.472289

-0.204078 -1.231778 0.228343

2.054522 0.059492 0.344428

2.541950 0.956923 -0.028017

1.852877 0.127306 1.411177

2.639777 -0.826489 0.111504

-0.095655 1.272278 0.128552

-1.051780 1.294419 -0.057777

-2.692381 0.059848 0.114874

-2.051653 -0.669386 0.279030

**TS4-1**

-0.180515 0.140969 -0.195602

-0.121311 1.326970 0.654988

-1.917637 -0.188723 -0.469316

-2.022579 -1.110412 -1.034663

-2.377753 -0.268602 0.513623

-2.317537 0.659763 -1.017453

0.028693 -1.195513 0.698904

0.986407 -1.345146 0.725263

2.315112 -0.116034 -0.482857

2.345573 0.817855 -0.209524

**C4-2**

0.006016 -0.133240 -0.158530

-0.045876 -1.275062 0.729323

1.856456 0.066097 -0.424079

2.018090 0.929253 -1.061604

2.289656 0.194892 0.564491

2.177422 -0.858549 -0.892285

-0.048869 1.276242 0.599086

-0.981522 1.481671 0.761234

-1.737392 0.096282 -0.529027

-2.081540 -0.791710 -0.685934

**TS4-2**

-0.092991 -0.133110 -0.200534

-0.123794 -1.230350 0.741448

2.111892 0.003755 -0.359891

2.282330 0.876766 -0.975232

2.353023 0.103771 0.689953

2.344396 -0.951897 -0.809750

-0.024974 1.290123 0.545069

-0.907828 1.501856 0.883181

-1.739340 0.108029 -0.524868

-2.150558 -0.765687 -0.513458

**H_2_SO_3_**

-0.000001 0.110285 -0.435214

0.000028 1.354649 0.310614

-1.236611 -0.738371 0.151399

-1.469764 -0.393721 1.028078

1.236583 -0.738414 0.151397

1.469776 -0.393745 1.028058

**CH_3_**

0.000004 -0.000183 -0.000390

-0.929659 -0.542714 0.000780

0.935547 -0.532499 0.000780

-0.005910 1.076313 0.000779

**TS**

-0.118437 -0.149093 -0.440250

-0.715990 -1.219476 0.362803

1.534633 0.093395 0.204190

1.939028 1.019484 -0.197831

1.454077 0.130686 1.288351

2.130744 -0.758424 -0.116045

-0.699808 1.267841 0.162574

-1.510268 1.046449 0.641373
